# Effect of Angelica polysaccharide on mouse myeloid-derived suppressor cells

**DOI:** 10.3389/fimmu.2022.989230

**Published:** 2022-09-09

**Authors:** Jie Shen, Mengyu Zhang, Ke Zhang, Yahan Qin, Meifang Liu, Shujuan Liang, Daquan Chen, Meiyu Peng

**Affiliations:** ^1^ Weifang Medical University, Weifang, China; ^2^ Key Lab for Immunology in Universities of Shandong Province, Department of Immunology, School of Basic Medical Sciences, Weifang Medical University, Weifang, China; ^3^ Collaborative Innovation Center of Advanced Drug Delivery System and BiotechDrugs, School of Pharmacy, Yantai University, Yantai, China

**Keywords:** Angelica polysaccharide, myeloid-derived suppressor cells, mannose receptor, proliferation, differentiation, immunosuppression

## Abstract

Angelica polysaccharide (APS) is a polysaccharide extracted from Angelica sinensis and it is one of the main active components of Angelica sinensis. Many studies have demonstrated that APS can promote the activation and function of a variety of immune cells and is recognized as an immune enhancer, but the regulatory effect of APS on myeloid-derived suppressor cells (MDSC) is still unclear. In this study, we investigated the effects of APS on MDSC proliferation, differentiation and function through *in vivo* and *in vitro* experiments. *In vitro*, our results showed that APS promoted the proliferation, differentiation and immunosuppressive function of MDSC through STAT1 and STAT3 signaling pathways, and positively correlated with the expression level of Mannose receptor (MR, also known as CD206) and in a concentration-dependent manner on APS. *In vivo*, APS up-regulated T cells, γδT cells, CD8^+^T cells, natural killer cells, monocytes/macrophages, and granulocytes in the peripheral blood and spleen of mice to varying degrees and was accompanied by the same degree of increase in the proportion of MDSC. That reminds to the clinician that when applying APS as treatment they should pay attention to its possible side effects of increasing the quantity and function of MDSC, in order to increase its efficacy.

## Introduction

Angelica polysaccharide (APS) is a polysaccharide extracted from Angelica sinensis and is one of the main active components of Angelica sinensis. It retains the effects of Angelica sinensis against radiation, has anti-tumor effects and low side effects, promotes bone marrow hematopoiesis, accelerates blood circulation and enhances cell mediated immunity and humoral immune response (1–3). Numerous studies have demonstrated that APS protects a variety of immune cells, promotes their proliferation and differentiation, and induces their maturation *in vitro* and *in vivo*.

APS can act directly on macrophages, promote macrophage proliferation and significantly improve their phagocytic ability (4, 5). Activated macrophages release large amounts of effector molecules such as Nitrous oxide (NO), tumor necrosis factor (TNF)-α and reactive oxygen species (ROS), which increase the activity of lysozyme (6). APS can also increase the amount of blood calcium in the body, which in turn protects immune cells and enhances the phagocytic function of macrophages. Direct exposure of APS to splenocytes, increased the expression levels of interleukin-2 (IL-2), interleukin-4 (IL-4), interleukin-6 (IL-6), and interferon (IFN)-γ in macrophages, helper T cell (Th) cells, and NK cells. In addition, APS is also a mitogen of B cells, stimulating B lymphocytes to differentiate into intermediate stages that can respond to helper T lymphocytes and accelerating the process of humoral immunity (7). *In vitro* mixed lymphocyte reaction demonstrated that APS could promote the response of mouse splenocytes to allotype antigens and significantly promote the proliferation of mixed lymphocytes and the secretion of IFN-γ, showing a strong immune-promoting effect (8, 9). APS is also a potential mitogen for mouse T cells and plays an immune regulatory role by regulating the expression of Th1 and Th2 related cytokines (10). APS has a significant stimulating effect on major histocompatibility complex (MHC)- II, CD80, and CD86 molecules and can induce the differentiation and maturation of dendritic cells (DC) (11).

Although many studies have demonstrated that APS can promote the activation and function of a variety of immune cells and is recognized as an immune enhancer, the regulatory effect of APS on myeloid-derived suppressor cells (MDSC) is still unclear. MDSC, composed of precursor cells of granulocytes, macrophages, DC, and myeloid progenitor cells, is a collective term for a group of cells with immunosuppressive functions and has a strong ability to inhibit a variety of cell functions (12). In mice, MDSC are mainly divided into two major categories: CD11b^+^Ly6C^+/high^Ly6G^-^ monocytic MDSC (M-MDSC) and CD11b^+^Ly6C^low^Ly6G^+^ granulocytic MDSC (PMN-MDSC) (13).

In this study, we aimed to investigate the effects of APS on MDSC proliferation, differentiation and function through *in vivo* and *in vitro* experiments and explore its molecular mechanism. *In vitro*, we isolated bone marrow cells (BMC) from normal mice to induce differentiation of MDSC, and observed the effect of APS on MDSC proliferation and differentiation. We also isolated MDSC from tumor-bearing mice to observe the effect of APS on MDSC function and its molecular mechanism. In addition we observed the expression level of Mannose receptor (MR, also known as CD206) in BMC and MDSC. *In vivo*, we observed the changes in the proportion of immune cells in mice after APS gavage treated. Our results showed that APS promoted proliferation, differentiation, and immunosuppressive function of MDSC *via* STAT1 and STAT3 signaling pathways *in vitro* and correlated with the expression level of MR (CD206) in a concentration-dependent manner. In addition, APS can up-regulate the numbers of MDSC and other immune cells in peripheral blood and spleen of mice to varying degrees *in vivo*.

## Materials and methods

### Animal and tumor models

Specific pathogen free (SPF) grade 6 to 8 week old female C57BL/6 mice (purchased from Weifang Medical University Laboratory Animal Breeding Center, No.370843220100000932) were housed in independent individual ventilated cages (IVC) of the animal room. Mice were acclimated to the environment for 7 days prior to the start of the experiment. All animal experiments were performed in accordance with the animal ethics requirements of Weifang Medical University.

Mouse pancreatic cancer cell line H7 cells were cultured in Dulbecco’s modified Eagle’s medium (DMEM) containing 100 U/ml penicillin streptomycin mixture (Solarbio, China) and 10% fetal bovine serum (FBS) in an incubator with 5% CO_2_ at 37°C. Log phase H7 cells were collected and subcutaneously inoculated at a dose of 1×10^6^ cells/point to establish a mouse tumor model.

### Acquisition of bone marrow cells

Normal mice were sacrificed by CO_2_ asphyxiation and soaked in 75% alcohol for 3-5 min. The lower limb bones of mice were aseptically extracted in a super clean bench. The abdominal skin between the hips of the mice was pinched with ophthalmic forceps, the skin was carefully cut with ophthalmic scissors, and the skin of the lower limbs was separated, cut at the ankle inferiorly and the hip superiorly. The two lower limbs of the mice were freed. Muscle and connective tissue were carefully dissected, femur and tibia were dissected, and cartilage was cut off at both ends to expose the red marrow cavity. PBS was aspirated with a sterile 1 ml syringe, gently inserted into the bone marrow cavity, and the bone marrow cavity was repeatedly flushed to obtain bone marrow cells (BMC).

### Acquisition of MDSC

*In vitro*, MDSC were induced for differentiation from BMC: BMC were isolated from normal mice, adjusted to a cell concentration of 1×10^6^ cells/ml, placed in 12-well plates, 2 ml per well of complete 1640 medium, then cells were cultured with recombinant mouse GM-CSF (Sigma-Aldrich, USA, 1 μl/ml) and APS (Youke, China, purity ≥ 98%) at different concentrations (0, 160 μg/ml) for 4 days, half changed every two days.

Ex *vivo*, MDSC were isolated from normal or tumor-bearing mice: Peripheral blood was collected from the heart, BMC and spleens were collected from normal or tumor-bearing mice, and single splenocytes were prepared by grinding and filtering with a 40 μm strainer. Following hemolysis, all cells were mixed and re-suspended with an equal volume of 1 bead enrichment. Anti-mouse Ly6C and Ly6G beads (BD Biosciences, USA) were added to 10 μl/10^7^ cells at 6-12°C for 15 min, after which unbound cells were washed off with 20-fold 1 bead enrichment solution (BD Biosciences, USA) and the supernatant was discarded by centrifugation. Two milliliters of 1 bead enrichment solution were used to re-suspend the cells, transferred into a sorting tube, placed in a magnetic stand, and the supernatant was carefully aspirated. Sorted MDSC were re-suspended with complete RPMI 1640 medium, that was supplemented with glutamine (200 mmol/L), penicillin streptomycin (100 U/ml) and 10% FBS.

### Detection of MR (CD206) expression on BMC and MDSC

BMC were isolated from normal mice (on day 0), and cultured with recombinant mouse GM-CSF in complete RPMI 1640 medium for 4 days (on day 4) for defferentiation of MDSC. MDSC induced *in vitro* from BMC were collected and blocked with Anti-Mouse CD16/32 antibody (Biolegend, USA) for 15 min, and then stained with fluorescence-labeled antibody Anti-Mouse CD11b-FITC (BD Biosciences, USA) or Anti-Mouse CD11b-BV421 (BD Biosciences, USA), in the dark at 4°C for 30 min, followed adding Fixation and Permeabilization buffer (BD Biosciences, USA, 250 μl/tube), at 4°C for 20 min. Cells were washed with 1 ml of 1×Perm/Wash™ buffer (BD Biosciences, USA), and finally, Anti-Mouse CD206-AF647 (BD Biosciences, USA) or Anti-Mouse CD206-BV421 (Biolegend, USA) was added and incubated at 4°C for 30 min in the dark and detected by BD FACSVerse flow cytometer and quantified with FlowJo 7.6 software.

### Detection of MDSC proliferation

BMC isolated from normal mice were labeled with CFSE (Thermo, USA), adjusted to a cell concentration of 1×10^6^ cells/ml, and cultured in 12-well plates at 2 ml per well in complete RPMI 1640 medium, mixed with APS (Youke, China, purity ≥ 98%) at different concentrations (0, 80, 160, and 320 μg/ml) for 72 h, with or without recombinant mouse GM-CSF (Sigma-Aldrich, USA, 1 μl/ml). Finally, the cells were assessed using BD FACSVerse flow cytometer and quantified with FlowJo 7.6 software.

### Detection of MDSC differentiation

MDSC induced *in vitro* from BMC, were collected and stained with fluorescence-labeled antibodies Anti-Mouse CD11b-FITC, Anti-Mouse Ly6C-APC, and Anti-Mouse Ly6G-PE (BD Biosciences, USA), and the proportions of M-MDSC, PMN-MDSC, and total MDSC cells were measured by BD FACSVerse flow cytometer and quantified with FlowJo 7.6 software.

### ROS and H_2_O_2_ detection

MDSC isolated from tumor-bearing mice were cultured with recombinant mouse GM-CSF (Sigma-Aldrich, USA, 1 μl/ml) and APS (Youke, China, purity ≥ 98%) at different concentrations (0, 80, 160, and 320 μg/ml) for 48h. ROS activity was detected using an intracellular ROS kit (Abcam, USA) and H_2_O_2_ concentration was detected using an intracellular hydrogen peroxide detection kit (Abcam, USA) according to the manufacturer’s instruction. Finally, the cells were assessed using BD FACSVerse flow cytometer and quantified with FlowJo 7.6 software.

### Quantitative real-time PCR

MDSC isolated from tumor-bearing mice were cultured with recombinant mouse GM-CSF (Sigma-Aldrich, USA, 1 μl/ml) and APS (Youke, China, purity ≥ 98%) at different concentrations (0, 160 μg/ml) for 24h. RNA was extracted by TRIzol reagent, and the first-strand cDNA synthesis was performed following the reverse transcription kit operating instructions. GAPDH was used as an internal control. Primer sequences used for qPCR were as follows: ARG-1, (F) 5’-TGTCCCTAATGACAGCTCCTT-3’ and (R) 5’-GCATCCACCCAAATGACACAT-3’; iNOS, (F) 5’-GCCACCAACAATGGCAACAT-3’ and (R) 5 ‘-TCGATGCACAACTGGGTGAA-3’; GAPDH, (F) 5’-TGTCTCCTGCGACTTCAACA-3’ and (R) 5 ‘-GGTGGTCCAGGGTTTCTTACT-3’. Experiments were completed on a Roche Light Cycler 480 fluorescence quantitative PCR instrument. The data obtained from the experiment were calculated according to the 2^-ΔΔCt^ method and statistically analyzed.

### Intracellular signal molecules were detected by flow cytometry

MDSC isolated from tumor-bearing mice were cultured with recombinant mouse GM-CSF (Sigma-Aldrich, USA, 1 μl/ml) and APS at different concentrations (0, 80, 160, 320 μg/ml) for 12h. Collected cells were fixed and permeated with Fixation and Permeabilization buffer (BD Biosciences, USA, 250 μl/tube) at 4°C for 20 min, and then washed with 1 ml of 1×Perm/Wash™ (BD Biosciences, USA); blocked with Anti-Mouse CD16/32 (Biolegend, USA) at 4°C for 15 min; and incubated with Anti-Mouse fluorescence-labeled phosphorylated antibodies STAT1-AF647 (p-STAT1) and phosphorylated antibodies STAT3-FITC (p-STAT3) (Biolegend, USA) at 4°C for 30 min in the dark. Finally, the cells were assessed using BD FACSVerse flowcytometer and quantified with FlowJo 7.6 software. The isotype controls were used to adjust the instrument.

### The effect of APS on the number of immune cells *in vivo* was detected

APS (Youke, China, purity ≥ 98%) was prepared with double distilled water (DW). Mice in the experimental group (n=6) were given 200 mg/kg APS by gavage once daily for 8 weeks. The control group (n=6) was treated with the same amount of DW. At the end of intragastric administration, the mice were sacrificed, the body weights, spleen and thymus weights were measured, peripheral blood was collected from the heart and spleen samples were collected, and splenocyte single cells were prepared by grinding and filtering with a 40 μm mesh screen, and dissolving red blood cells. The cell concentration was adjusted to 1×10^6^ cells/mL, and 100 μl of cells were added to each detection tube.

The fluorescence-labelled antibodies added in detection groups were as follows. (1) T cells and subpopulations detection: Anti-Mouse CD3-Percp (Biolegend, USA), Anti-Mouse CD4-APC-H7, Anti-Mouse CD8-APC (BD Biosciences, USA) and Anti-Mouse γδ-PE (Invitrogen, OR, USA) antibodies; (2) B cells and NK cells detection: Anti-Mouse CD19-APC (BD Biosciences, USA) and Anti-Mouse NK1.1-BV421 (Biolegend, USA) antibodies; (3) monocyte/macrophage detection: Anti-Mouse CD11b-FITC and Anti-Mouse F4/80-PE (BD Biosciences, USA) antibodies; (4) MDSC detection: Anti-Mouse CD11b-FITC (BD Biosciences, USA), Anti-Mouse Ly6C-APC and Anti-Mouse Ly6G-PE (Biolegend,USA) antibodies; (5) Granulocyte detection: Anti-Mouse CD11b-FITC (BD Biosciences, USA) and Anti-Mouse Gr1-APC (Biolegend, USA) antibodies. Cells of above groups (1) to (5) were incubated at 4°C for 30 min in the dark and detected by flow cytometry. (6)Treg cells detection: Anti-Mouse CD4-APC-H7 (BD Biosciences, USA) and Anti-Mouse CD25-APC (Invitrogen, OR, USA) antibodies. Cells were incubated in the dark at 4°C for 30 min, and Fixation and Permeabilization buffer (BD Biosciences, USA, 250 μl/tube) was used to fix and permeate at 4°C for 20 min. Cells were washed with 1 ml of 1×Perm/Wash™ (BD Biosciences, USA), blocked with Anti-Mouse CD16/32 (Biolegend, USA) antibody for 15 min, Anti-Mouse Foxp3-PE (Invitrogen, OR, USA) antibody was added, and incubated in the dark at 4°C for 30 min. Finally, the cells were assessed using BD FACSVerse flow cytometer and quantified with FlowJo 7.6 software.

### Statistical analysis

The data represent the mean values ± the standard deviation (SD). Comparisons between the groups were performed using Student’s unpaired t-test; *p*-value<0.05 was considered statistically significant.

## Results

### The expression of MR (CD206) on MDSC and BMC

Some research found that MR (CD206) can bind to a variety of oligosaccharides and polysaccharides and initiate immune responses (14, 15). So, we first detected the expression of MR (CD206) on MDSC which were isolated from normal mice, MDSC which were differentiated *in vitro*, BMC which were isolated from mouse (in day 0), and BMC which were cultured with recombinant mouse GM-CSF in day 4.

Our results indicate there were some differences of MR (CD206) expression on M-MDSC and PMN-MDSC isolated from normal mice between different mouse individuals. Most mice M-MDSC (from 80.30% to 13.70%) (**Supplementary Figures A, B**) have lower MR (CD206) levels compared with PMN-MDSC (from 98.70% to 4.29%) (**Supplementary Figures C, D**).

Similarly, there were also some differences of MR (CD206) expression between M-MDSC and PMN-MDSC which were differentiated *in vitro* between different mouse individuals. The difference is that most mice M-MDSC (from 31.80% to 7.97%) (**Supplementary Figures E, F**) have higher MR (CD206) levels compared with PMN-MDSC (from 13.60% to 0.80%) (**Supplementary Figures G, H**). The MR (CD206) expression levels of both M-MDSC and PMN-MDSC (**Supplementary Figures F, H**) induced *in vitro* were significantly lower compared to MDSC which were isolated *in vivo* (**Supplementary Figures B, D**).

Moreover, the expression levels of MR (CD206) on isolated from mouse (in day 0) CD11b^+^ BMC varied also greatly between different mouse individuals (from 98.00% to 20.40%) (**Supplementary Figures I, J**). Moreover, the expression levels of MR (CD206) on CD11b^+^ BMC which were cultured with recombinant mouse GM-CSF in day 4 were significantly reduced (from 23.20% to 4.33%) (**Supplementary Figures K, L**).

These results mean that BMC and MDSC have higher expression of MR (CD206) *in vivo*.

Considering individual differences in MR (CD206) expression, we used the average MR (CD206) value of BMC which were isolated from 10 normal mice (in day 0) (60.09%) as a boundary to divide them into two groups with high MR (CD206) expression and low MR (CD206) expression. Then, the effects of APS on cell proliferation and differentiation from the two groups were examined when they were induced to differentiate into MDSC *in vitro*.

### Effect of APS on BMC and MDSC proliferation

It has been reported that APS has a proliferative and differentiating effect on human granulocytic hematopoietic progenitor cells in the presence of exogenous GM-CSF (16). So, we first detected the effect of APS on mouse BMC and MDSC proliferation in the absence and presence of exogenous GM-CSF.

**Figure 1:**
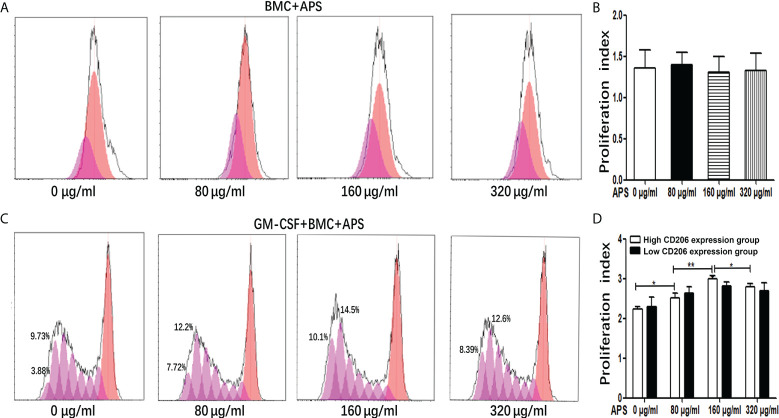
The effect of APS on MDSC proliferation. **(A)** BMC which were isolated from normal mouse were treated with APS (0, 80, 160 and 320 μg/ml) for 72 h. One representative result from each experiment is shown. **(B)** Proliferation index of BMC which were isolated from normal mouse treated with APS. **(C)** BMC which were isolated from normal mouse were treated with GM-CSF and APS (0, 80, 160 and 320 μg/ml) for 72 h. One representative result from each experiment is shown. **(D)** Proliferation index of MDSC (^**^p<0.01, ^*^p<0.05). n=5, Data are expressed by mean ± SD.

As shown in **Figures 1A, B**, in the absence of exogenous GM-CSF, compared with the group without APS, the proliferation index of BMC had not obviously change with the increase of APS concentration (from 80 μg/ml to 320 μg/ml) (1.35 vs 1.39 vs 1.31 vs 1.33, p > 0.05). It means that APS had no effect on BMC proliferation.

As shown in **Figures 1C, D**, when BMC were induced to differentiate into MDSC by the addition of GM-CSF, in the high MR (CD206) expression group, compared with the group without APS, the proliferation index of MDSC gradually increased with the increase of APS concentration (from 80 μg/ml to 160 μg/ml) (2.24 vs 2.52 vs 3.00, p< 0.001). However, compared with the 160 μg/ml APS group, the 320 μg/ml APS treatment group showed a slight downward trend (3.00 vs 2.80, p< 0.05).

However, in the low MR (CD206) expression group, APS had no significant effect on the proliferation index of MDSC (2.30 vs 2.63 vs 2.82 vs 2.70, p > 0.05) (**Figure 1D**).

These results indicated that the promoting effect of APS on MDSC proliferation was positively correlated with the expression of MR (CD206) and in a concentration-dependent manner on APS.

### Effect of APS on MDSC differentiation

**Figure 2:**
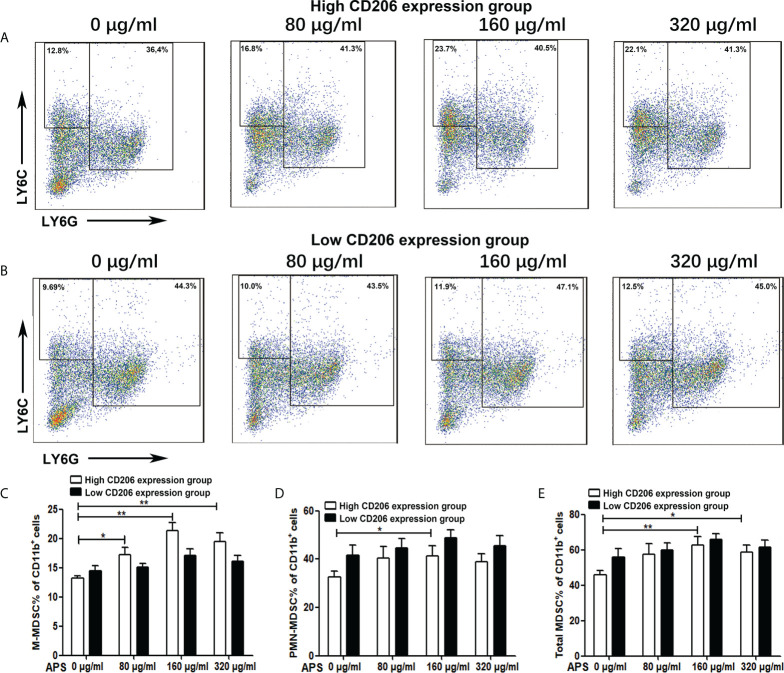
The effect of APS on MDSC differentiation. BMC which were isolated from normal mouse and were divided into the MR (CD206) high expression group and the MR (CD206) low expression group. Then they were treated with APS (0, 80, 160 and 320 μg/ml) for 96h in the presence of GM-CSF. **(A, B)** One representative result from each experiment is shown. The percentages of M-MDSC **(C)**, PMN-MDSC **(D)** and total MDSC **(E)** among the induced MDSC *in vitro* (^**^p<0.01, ^*^p<0.05). n=5, Data are expressed by mean ± SD.

Then we detected the effect of APS on mouse MDSC differentiation in the presence of exogenous GM-CSF. As shown in **Figures 2A, C–E**, in the high-expression MR (CD206) group, compared with the group without APS, the proportion of M-MDSC (13.24% vs 17.16% vs 21.30%, p< 0.01) (**Figure 2C**), PMN-MDSC (32.50% vs 40.26% vs 42.24%, p< 0.05) (**Figure 2D**), and total MDSC (45.74% vs 57.42% vs 63.54%, p< 0.01) (**Figure 2E**) differentiated from BMC gradually increased with increasing APS concentrations (from 80 μg/ml to 160 μg/ml). However, the proportion of M-MDSC (21.30% vs 19.50%) (**Figure 2C**), PMN-MDSC (42.24% vs 39.04%)(**Figure 2D**), and total MDSC (63.54% vs 58.54%) **(** **Figure 2E** **)** in the 320 μg/ml APS treatment group showed a slight decreasing trend compared with the 160 μg/ml APS group.

As shown in **Figures 2B, C–E**, in the low MR (CD206) expression group, APS had no significant effect on the proportion of M-MDSC (14.50% vs 15.14% vs 17.14% vs 16.12%, p > 0.05) (**Figure 2C**), PMN-MDSC (41.50% vs 44.60% vs 48.76% vs 45.44%, p > 0.05) (**Figure 2D**), and total MDSC (56.00% vs 59.74% vs 65.90% vs 61.56%, p > 0.05) (**Figure 2E**) differentiated from BMC of mice.

These results indicate that the promoting effect of APS on MDSC proliferation and differentiation was also positively correlated with the expression of MR (CD206) and in a concentration-dependent manner on APS.

### Effect of APS on MDSC function

It has been reported that MDSC inhibit T cell functions *via* multiple pathways, including the up-regulation of ROS, H_2_O_2_, arginase-1 (Arg-1) and iNOS production. We thus detected the ROS and H_2_O_2_ concentration and the expression levels of ARG-1 mRNA, iNOS mRNA of Ly6G^+^Ly6C^+^ MDSC which were sorted from tumor-bearing mice, as described in the Materials and Methods section.

**Figure 3:**
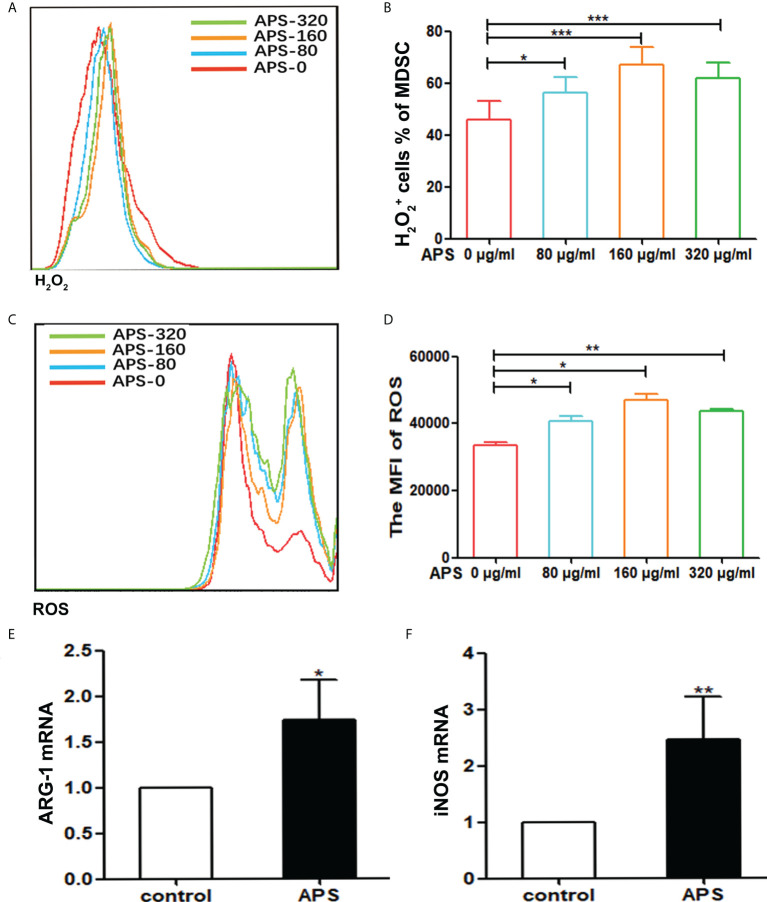
APS increases H_2_O_2_, ROS production and the expression of MDSC-associated genes. MDSC were isolated from peripheral blood, spleen and bone marrow from tumor-bearing mice using anti-mouse Ly-6G and Ly-6C particle-DM, then they were treated with APS (0, 80, 160 and 320 μg/ml) for 48h. **(A, C)** One representative result from each experiment is shown. The H_2_O_2_^+^ cells% **(B)** and the MFI of the ROS detection **(D)** in the isolated MDSC (*** p<0.001, ** p<0.01, * p<0.05). The expression of the MDSC associated genes ARG-1 **(E)** and iNOS **(F)** was analyzed using qPCR (** p<0.01, * p<0.05). n=5, Data are expressed by mean ± SD.

Compared with the group without APS, both H_2_O_2_ and ROS released from MDSC sorted from tumor-bearing mice increased significantly with increasing concentrations of APS added (from 80 μg/ml to 160 μg/ml). The proportion of MDSC expressing H_2_O_2_ was (46.00% vs 56.38% vs 67.13%, p< 0.001) (**Figures 3A, B**), and the mean fluorescence intensity (MFI) of ROS was (3.34×10^4^ vs 4.08×10^4^ vs 4.69×10^4^, p< 0.05) (**Figures 3C, D**). However, compared with the 160 μg/ml APS group, both H_2_O_2_ and ROS showed a slight decreasing trend in the 320 μg/ml APS treatment group, with values of (67.13% vs 61.78%)(**Figure 3B**) and (4.69×10^4^ vs 4.38×10^4^) (**Figure 3D**).

Similarly, compared with the group without APS, 160 μg/ml APS significantly increased the expression levels of ARG-1 mRNA (average fold-change 1.65, p< 0.05) (**Figure 3E**) and iNOS mRNA (average fold-change 2.45, p< 0.01) (**Figure 3F**) in MDSC.

These results indicated that APS could promote the immunosuppressive ability of MDSC and in a concentration-dependent manner.

### Effects of APS on spleen index and thymus index

At present, APS is a widely used drug that can boost the body’s immunity. However, our experiments showed that APS markedly upregulated the quantity and function of MDSC *in vitro*. Therefore, we examined the immune-potentiation of APS and the overall effect of the immunosuppressive up-regulation of MDSC *in vivo*.

**Figure 4:**
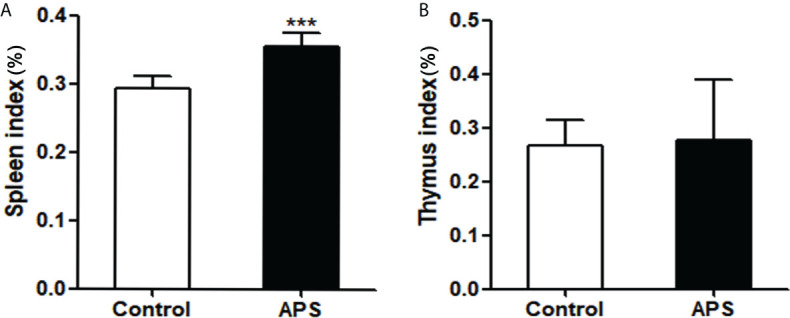
The effect of APS in spleen index and thymus index on mice. Mice were euthanized after 8 weeks of Control/APS-treated. **(A)** Final Spleen index (^***^p<0.001). **(B)** Final Thymic index. n=6, Data are expressed by mean ± SD.

First, we detected the effects of APS on spleen and thymus in mice. Compared with the control group, the spleen index (0.29% vs 0.36%, p< 0.001) was significantly increased in APS-treated mice (**Figure 4A**), but the thymus index (0.27% vs 0.28%, p > 0.05) was not significantly changed (**Figure 4B**).

These results showed that APS can strengthen the mouse organic immunity.

### Effects of APS on T cells, T subpopulations and B cells *in vivo*

To explore the effect of APS on adaptive immunity in mice, we detected the effects of APS on the quantity of T cells, T subpopulations and B cells *in vivo*.

**Figure 5:**
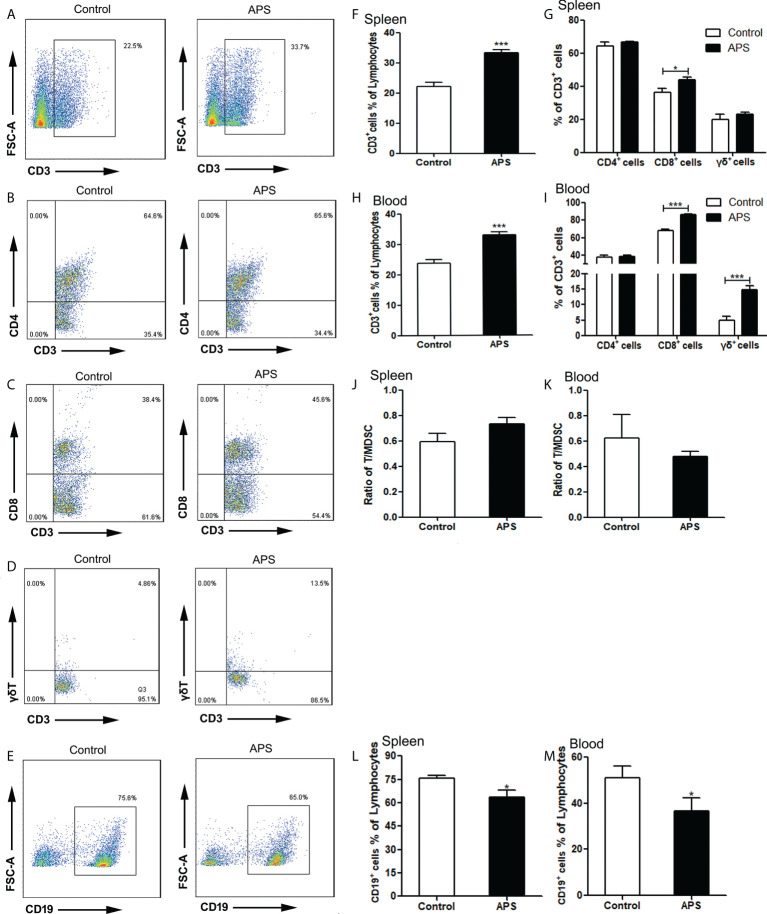
The percentages of T cells, T subpopulations and B cells in the spleen and peripheral blood. The spleen and peripheral blood were collected from the Control/APS-treated mice. Single-cell suspensions were generated, and the cells were immune-stained for CD3, CD8, CD4 and γδ. **(A–E)** One representative result from each experiment is shown. The percentages of CD3^+^ cells among the lymphocytes in spleen **(F)** and blood **(H)** (^***^p<0.001). The percentages of CD3^+^CD4^+^T, CD3^+^CD8^+^T cells and γδ^+^T cells among the CD3^+^T population from lymphocytes in spleen **(G)** and blood **(I)** (^***^p<0.001, ^*^p<0.05).The ratio of T/MDSC in splenocytes **(J)** and blood **(K)**. The percentages of CD19^+^ cells among the lymphocytes from splenocytes **(L)** and blood **(M)** (^*^p<0.05). n=6, Data are expressed by mean ± SD.

Compared with the control group, the proportion of splenic CD3^+^T cells was significantly increased in APS-treated mice (22.10% vs 33.24%, p< 0.001) (**Figures 5A, F**). Among them, CD8^+^T cells were significantly increased (36.46% vs 43.98%, p< 0.05) (**Figures 5C, G**), but not CD4^+^T cells (64.46% vs 66.50%, p > 0.05) (**Figures 5B, G**) and γδT cells (19.81% vs 22.94%, p > 0.05) (**Figure 5G**).

Compared with the control group, the proportion of peripheral blood CD3^+^T cells was significantly increased in APS-treated mice (23.77% vs 33.03%, p< 0.001) (**Figure 5H**). Among them, CD8^+^T cells (67.77% vs 86.35%, p< 0.001) (**Figure 5I**) and γδT cells (4.84% vs 14.83%, p< 0.001) (**Figures 5D, I**) were significantly increased, while CD4^+^T cells (37.75% vs 38.30%, p > 0.05) (**Figure 5I**) had no significant changes.

In addition, we compared the ratio of T cells to MDSC in the spleen (0.59 vs 0.74, p > 0.05) (**Figure 5J**) and peripheral blood (0.62 vs 0.48, p > 0.05)(**Figure 5K**), and there were no significant changes.

Compared with the control group, the proportion of both splenic B cells (75.77% vs 63.37%, p< 0.05) (**Figures 5E, L**) and peripheral blood B cells (50.98% vs 36.76%, p< 0.05) (**Figure 5M**) were significantly decreased in APS-treated mice.

These results showed that APS can up-regulate the proportions of T cells, γδT cells, CD8^+^ T cells in the peripheral blood and spleen of mice to varying degrees. T cells rise to the same degree as MDSC, but APS down-regulates the proportion of B cells in the peripheral blood and spleen of mice to varying degrees.

### Effects of APS on NK Cells, granulocytes and monocytes/macrophages *in vivo*

To explore the effect of APS on innate immunity in mice, we detected the effects of APS on the quantity of NK cells, granulocytes and monocytes/macrophages *in vivo*.

**Figure 6:**
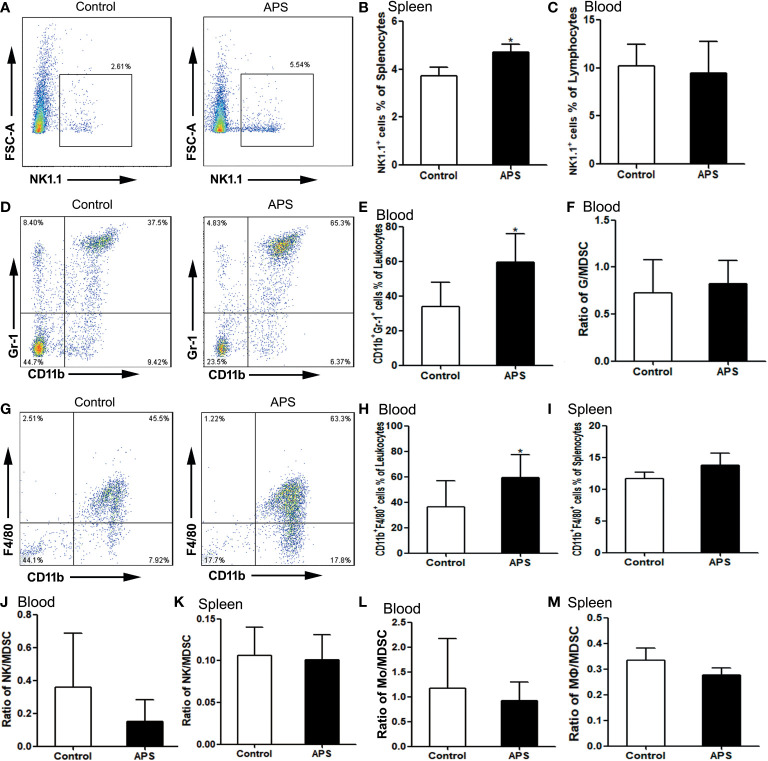
The percentages of NK cells, granulocytes and monocytes/macrophages in the spleen and peripheral blood. The spleen and peripheral blood were collected from the Control/APS-treated mice. Single-cell suspensions were generated, and the cells were immune-stained for CD19 and NK1.1. **(A, D, G)** One representative result from each experiment is shown. The percentages of NK1.1^+^ cells among the lymphocytes from splenocytes **(B)** and blood **(C)** (^*^p<0.05). **(E)** The percentages of CD11b^+^Gr1^+^ cells among the leukocytes in blood (^*^p<0.05). **(F)** The ratio of G/MDSC in blood. The percentages of CD11b^+^F4/80^+^ cells among the leukocytes in blood **(H)** and splenocytes **(I)** (^*^p<0.05). **(J)** The ratio of NK/MDSC in blood. **(K)** The ratio of NK/MDSC in spleen. **(L)** The ratio of Mo/MDSC in blood. **(M)** The ratio of Mɸ/MDSC in spleen. n=6, Data are expressed by mean ± SD.

Compared with the control group, NK cells were significantly increased in the spleen (3.73% vs 4.72%, p< 0.05) (**Figures 6A, B**), but not in the blood (10.20% vs 9.45%, p > 0.05) (**Figure 6C**). Meanwhile, we compared the ratio of NK to MDSC in peripheral blood (0.36 vs 0.15, p > 0.05) **(** **Figure 6J** **)** and spleen (0.11 vs 0.10, p > 0.05) **(** **Figure 6K** **)**, and there were no significant changes.

Compared with controls group, CD11b^+^Gr1^+^ granulocytes were significantly increased in the peripheral blood of APS-treated mice (33.98% vs 59.28%, p< 0.05) (**Figures 6D, E**). However, the ratio of granulocytes to MDSC did not change significantly (0.72 vs 0.83, p > 0.05) (**Figure 6F**).

Compared with the control group, peripheral blood CD11b^+^F4/80^+^ monocytes (36.32% vs 59.48%, p< 0.05) (**Figures 6G, H**) were significantly increased in APS-treated mice, while splenic CD11b^+^F4/80^+^ monocytes/macrophages (11.64% vs 13.79%, p > 0.05) (**Figure 6I**) were not significantly changed. Meanwhile, we compared the ratio of monocytes/macrophages to MDSC in peripheral blood (1.17 vs 0.92, p > 0.05) (**Figure 6L**) and spleen (0.33 vs 0.28, p > 0.05) (**Figure 6M**), and there were no significant changes.

These results showed that APS can up-regulate the proportions of NK cells, monocytes/macrophages and granulocytes in the peripheral blood and spleen of mice to varying degrees. Granulocytes, monocytes/macrophages also rise to the same degree as MDSC.

### Effect of APS on Treg and MDSC *in vivo*

To explore the effect of APS on immunosuppressive cells in mice, we detected the effects of APS on the quantity of Treg and MDSC *in vivo*.

**Figure 7:**
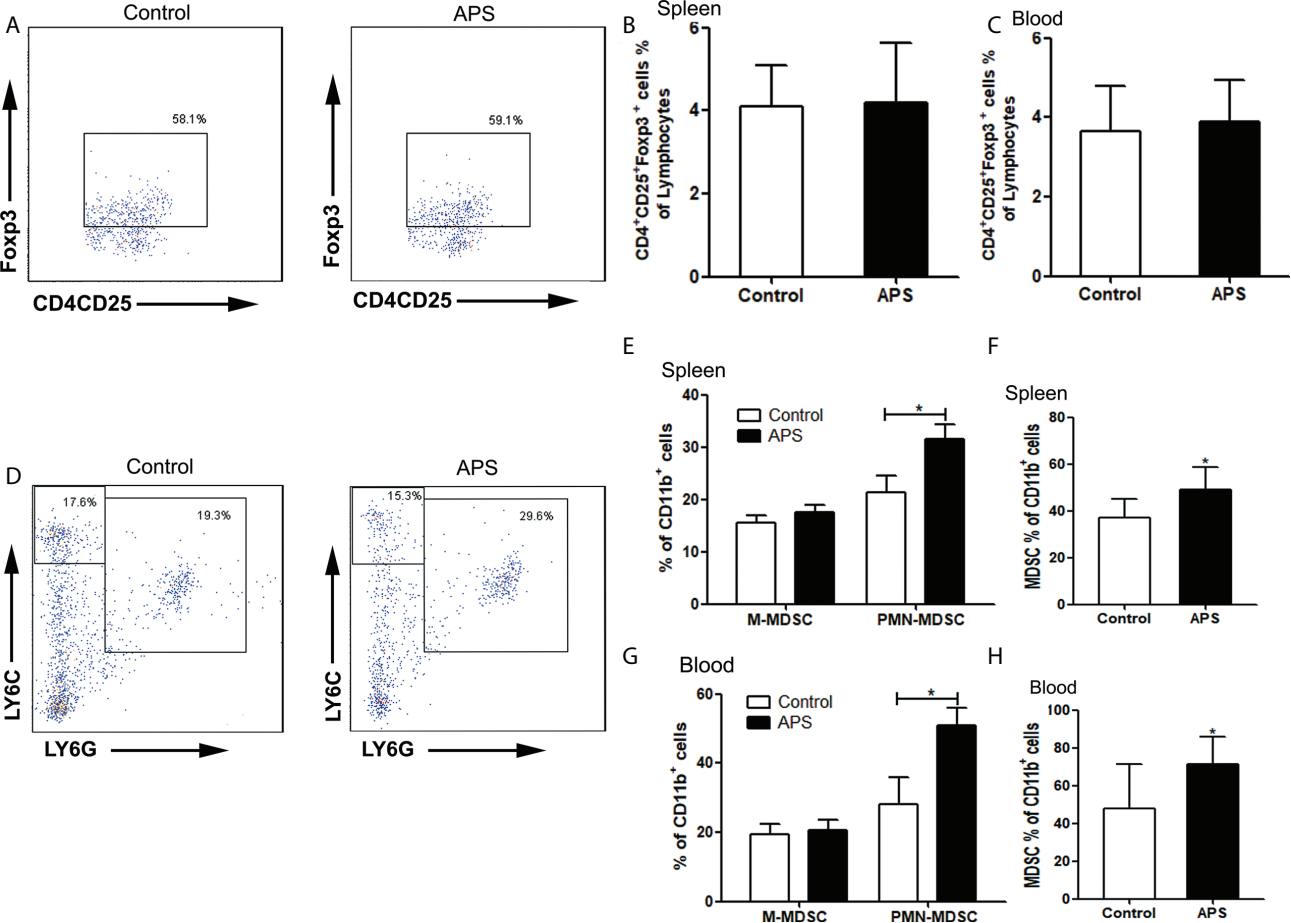
The percentages of Treg and MDSC in spleen and peripheral blood. The spleen and peripheral blood were collected from the Control/APS-treated mice. Single-cell suspensions were generated, and the cells were immune-stained for Treg、M-MDSC、PMN-MDSC and MDSC. **(A, D)** One representative result from each experiment is shown. The percentages of Treg cells among lymphocytes in spleen **(B)** and blood **(C)**. The percentages of M-MDSCs and PMN-MDSCs of CD11b^+^ cells among splenocytes **(E)** and leukocytes in blood **(G)** (^*^p<0.05). The percentages of total MDSC among splenocytes **(F)** and leukocytes in blood **(H)** (^*^p<0.05). n=6, Data are expressed by mean ± SD.

Compared with the control group, there were no significant changes in the proportions of splenic Treg cells (4.09% vs 4.20%, p > 0.05) (**Figures 7A, B**) and peripheral blood Treg cells (3.66% vs 3.89%, p > 0.05) (**Figure 7C**) in APS-treated mice.

Compared with the control group, the proportion of splenic PMN-MDSC (21.43% vs 31.62%, p< 0.05) (**Figures 7D, E**), total MDSC (36.88% vs 49.17%, p< 0.05) (**Figures 7D, F**) and peripheral blood PMN-MDSC (28.08% vs 50.75%, p< 0.05) (**Figure 7G**), total MDSC (47.78% vs 71.41%, p< 0.05) (**Figure 7H**) were significantly increased in APS-treated mice. However, there were no significant changes in the proportion of splenic M-MDSC (15.45% vs 17.55%, p > 0.05) (**Figures 7D, E**) and peripheral blood M-MDSC (19.32% vs 20.66%, p > 0.05) (**Figure 7G**).

These results showed that APS can up-regulate the proportions of MDSC in the peripheral blood and spleen of mice to varying degrees. But APS has no effect on Treg.

### Effects of APS on STAT1 and STAT3 signaling pathway of MDSC

To explore the mechanism by which APS promote the differentiation and immunosuppressive function of MDSC, we observed the effect of APS in the levels of p-STAT1 and p-STAT3 in MDSC which were sorted from tumor-bearing mice by flowcytometer. We analyzed the MFI (mean fluorescence intensity) of p-STAT1 or p-STAT3 among MDSC from the level of a single cell.

**Figure 8:**
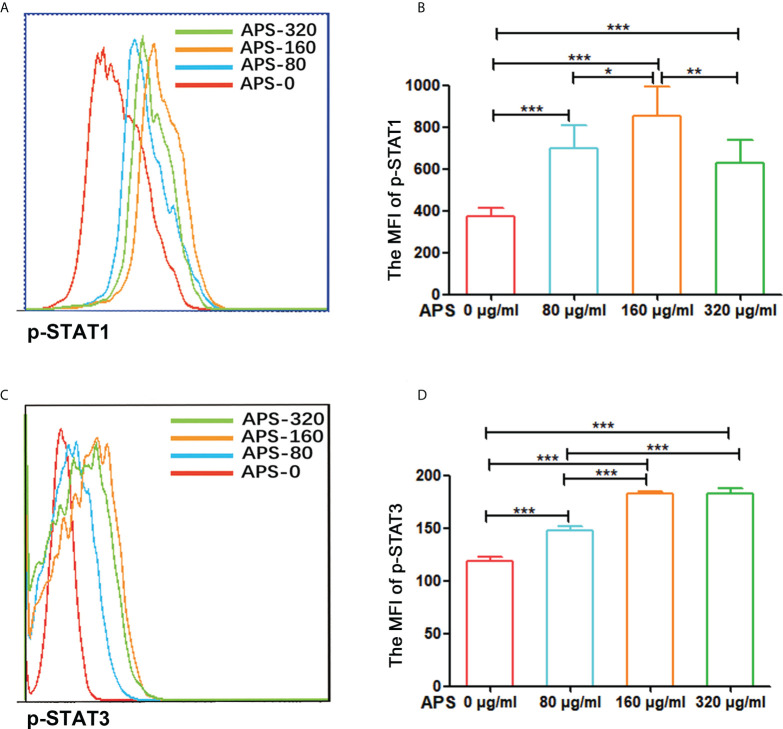
APS up-regulated STAT1 and STAT3 signaling pathways in MDSC. MDSC isolated from tumor-bearing mice were treated with APS at a concentration of 0, 80, 160 and 320 μg/ml for 12 h. **(A, C)** One representative result from each experiment is shown. **(B)** The MFI of p-STAT1 in MDSC (^***^p<0.001, ^**^p<0.01, ^*^p<0.05). **(D)** The MFI of p-STAT3 in MDSC (^***^p<0.001). n=6, Data are expressed by mean ± SD.

Compared with the group without APS, the expression levels of p-STAT1 and p-STAT3 in MDSC increased significantly with increasing APS concentrations (from 80 μg/ml to 160 μg/ml), with MFI values of (374.67 vs 696.50 vs 853.17, p< 0.001) (**Figures 8A, B**), and (119.00 vs 147.67 vs 182.50, p< 0.001), respectively (**Figures 8C, D**). However, compared with the 160 μg/ml APS group, 320 μg/ml APS treated group showed a certain downward trend in p-STAT1, with MFI values of (853.17 vs 630.17, p< 0.01) (**Figure 8B**); p-STAT3 did not change much (182.50 vs 182.33, p > 0.05) (**Figure 8D**).These results showed that APS markedly elevated the phosphorylation levels of STAT1, STAT3 in MDSC and in a dose-dependent manner.

## Discussion

APS is a water-soluble polysaccharide mainly composed of caramel, xylose, glucose, galactose, arabinose, and galacturonic acid, with multiple repeated “active centers” (17). Some studies had shown that the immune-pharmacological effects of angelica polysaccharide are achieved by recognizing a certain receptor and acting on a variety of immune effector cells to produce corresponding cytokines, which are involved in the nervous, humoral and endocrine systems of the body (4, 10). Mannose receptor (MR, also known as CD206) can specifically recognize and bind to ligands with mannose, N-acetylglucosamine or fucose as termini, mediating the recognition, phagocytosis and clearance of pathogens. Therefore, it is involved in the body’s innate versus acquired immunity (18, 19). It has been documented that MR (CD206) can bind to a variety of oligosaccharides and polysaccharides and initiate immune responses (14, 15). So, we first detected the expression of MR (CD206) in mouse MDSC which were sorted from normal mice, MDSC which were differentiated *in vitro*, BMC which were isolated from mouse (in day 0), and BMC which were cultured with recombinant mouse GM-CSF in day 4. As shown in **Supplementary Figure**, MR (CD206) was expressed in both mouse BMC and MDSC. However, its expression level varies greatly between different individual mouse cells. In addition, we found that compared with MDSC which were sorted from mouse, the MR (CD206) expression level of MDSC which were induced *in vitro* was significantly lower. Similarly, MR (CD206) expression levels were significantly lower in CD11b^+^ BMC which were cultured with recombinant mouse GM-CSF in day 4 compared with CD11b^+^ BMC which were isolated *in vivo* (in day 0). In fact, the most cells of CD11b^+^ BMC which were cultured with recombinant mouse GM-CSF after 4 day is MDSC. These results mean that BMC and MDSC have higher expression of MR (CD206) *in vivo*, while the mechanism needs to be further explored. In any case, we can speculate that MDSC induced *in vitro* are not exactly the same as the MDSC *in vivo* generated. So we used MDSC which were isolated from tumor-bearing mouse for subsequent experiments, including investigating the effects of APS on MDSC function and exploring its molecular mechanism.

Considering individual differences in MR (CD206) expression, we used the mean value of MR (CD206) expression levels in BMC which were isolated from (in day 0) 10 mice (60.09%) as a boundary and divided them into the MR (CD206) high expression group and the MR (CD206) low expression group. Experiments were performed to further explore whether the expression level of MR (CD206) correlates with the regulatory ability of APS on MDSC *in vitro*.

As shown in **Figures 1C, D**, when BMC were induced to differentiate into MDSC with GM-CSF *in vitro*, the cell proliferation index of BMC in the high MR (CD206) expression group gradually increased with the increase of APS concentration, and the effect was the best in the 160 μg/ml APS group. However, the proliferation index of BMC in the low MR (CD206) expression group did not change significantly. This indicated that the promoting effect of APS on MDSC proliferation was positively correlated with the expression of MR (CD206) and in a concentration-dependent manner on APS. But we found that APS has no effect on the proliferation of BMC in the absence of GM-CSF (**Figures 1A, B**). This is consistent with the report that APS has been found to have a proliferative and differentiating effect on human granulocytic hematopoietic progenitor cells only in the presence of exogenous GM-CSF (16). This suggests that APS has no effect on the proliferation of myeloid cells. The mechanism needs to be further explored.

Similarly, the proportion of total MDSC, M-MDSC, and PMN-MDSC induced from MR (CD206)-high expression group BMC also rose significantly in a concentration-dependent manner (**Figures 2C–E**). However, the degree of elevation of PMN-MDSC is not as obvious as that of M-MDSC. This may be associated with too low levels of MR (CD206) expression in PMN-MDSC induced *in vitro* (**Supplementary Figures F, H**). Similarly, there was no significant change in the proportion of MDSC induced from MR (CD206)-low expression group BMC (**Figures 2C–E**). These results indicate that the promoting effect of APS on MDSC differentiation was also positively correlated with the expression of MR (CD206) and in a concentration-dependent manner on APS.

MDSC can exert immunosuppressive functions through a variety of mechanisms, such as they can induce the production of NO and ROS, increase the proportion of Treg cells, increase arginase activity, and increase cysteine consumption (20). In terms of functional difference, both M-MDSC and PMN-MDSC exert inhibitory effects through arginase. Due to the high level of intracellular arginase in MDSC, L-arginine in the surrounding environment is depleted, which limits the formation of CD3ζ chains in T cells and so T cells arrest in the G0-G1 phase of the cell cycle (21). However, PMN-MDSC could up-regulate the activation of STAT3 and NADPH and affect the expression of ROS. Nitrate T cell receptors are induced by H_2_O_2_ and peroxynitrite to prevent the formation of bound peptide/MHC complexes (22, 23). Moreover, increased ROS production also contributes to the inhibition of differentiation of myeloid cells. In contrast, M-MDSC can activate STAT1, upregulate the expression of iNOS, and exert immunosuppressive functions (24). Our results showed that the levels of ROS and H_2_O_2_ released by MDSC (**Figures 3D, B**) gradually increased with the increase of APS concentration, and the 160 μg/ml APS group had the best effect, the expression levels of mRNA for ARG-1 and iNOS were also significantly increased after MDSC were treated with APS (**Figures 3E, F**). These results indicate that APS can promote the immunosuppressive ability of MDSC and in a concentration-dependent manner.

APS has the pharmacological effects of enhancing immune function and anti-tumor effects and is a widely used immune-enhancing drug at present. However, our experiments show that APS can markedly up-regulate the quantity and function of MDSC *in vitro*. Therefore, we examined the immune-potentiation of APS and the overall effect of the immunosuppressive up-regulation of MDSC by *in vivo* assays. Our results show that APS can increase the spleen index in mice, but has no effect on the thymus (**Figure 4**), which is consistent with reports from other studies (8). APS can up-regulate the proportions of T cells, γδT cells, CD8^+^T cells, NK cells, monocytes/macrophages, and granulocytes in the peripheral blood and spleen of mice to varying degrees (**Figures 5**, **6**), and it was accompanied by an increase in the proportion of total MDSC (**Figure 7**), with PMN-MDSC increasing more significantly, but there was no change in M-MDSC. This may be associated with a higher proportion of PMN-MDSC *in vivo* (**Figures 7E, G**), which can express higher levels of MR (CD206) compared to M-MDSC (**Supplementary Figures D, B**). We further compared the ratio of elevated T cells, NK cells, granulocytes and monocytes/macrophages to MDSC, and none of them changed significantly (**Figures 5J, K** and **Figures 6F, J–M** **).** This implies that APS is synchronously upregulating all immune cells *in vivo*. This provides an experimental basis for the clinical application of APS treatment and notes that APS may also have side effects by increasing the quantity and function of MDSC. In addition, our results show that APS can down-regulate the proportion of B cells *in vivo* (**Figures 5L, M**), with no effect on Treg cells (**Figures 7B, C**), and the mechanism involved needs to be further explored.

Both studies with melanoma patients *in vivo* (25) and with the multi-targeted tyrosine kinase inhibitor, sunitinib (26), demonstrated that STAT3 is essential for the proliferation of MDSC. The STAT3 signaling pathway can promote cell proliferation, reduces cell apoptosis, and prevents the differentiation of myeloid cells into mature cells by driving the expression of Bcl-xL, c-myc, and cyclin D1. It contributes to the mobilization, accumulation, and survival of MDSC. Similarly, the role of STAT1 in the function of M-MDSC is particularly important. STAT1 is involved in the regulation of arginase and iNOS activity and blocks IFN-γ secretion by T cells (27). Our results show that APS can markedly elevate the phosphorylation levels of STAT1, STAT3 in MDSC and in a dose-dependent manner (**Figure 8**). This suggests that APS up-regulates the proliferation, differentiation, and immunosuppressive functions of MDSC in a dose-dependent manner that may be associated with STAT1 and STAT3 signaling pathways.

## Conclusion

Our experiments show that APS has no effect on myeloid cells proliferation in the absence of GM-CSF. APS can promote the proliferation, differentiation and immunosuppressive function of MDSC through STAT1 and STAT3 signaling pathways, and it is positively correlated with the expression level of MR (CD206) and in a concentration-dependent manner on APS. These results suggest that clinical application of APS treatment should be careful because of its possible side effects of increasing the quantity and function of MDSC, in order to increase its efficacy.

## Data availability statement

The original contributions presented in the study are included in the article/**Supplementary Material**. Further inquiries can be directed to the corresponding authors.

## Ethics statement

The animal study was reviewed and approved by the animal ethics requirements of Weifang Medical University.

## Author contributions

JS and MZ performed the experiments and analysed the data. KZ and YQ established the animal models and collected samples. ML revised the article. MP, SL and DC designed the experiments and revised the article. All authors read and approved the final version of the manuscript.

## Funding

This work was supported by the National Natural Science Foundation of China through grant No. 81972695, 81873883, 82000525 and "Innovation Team of Immune Microenvironment and Inflammatory Diseases Research” of Introduction and Education Program for young Talents in Shandong Colleges and universities. Taishan Scholar Foundation of Shandong Province (No.qnts20161035);Natural Science Foundation of Shandong Province (No. ZR2019ZD24, ZR2019YQ30).

## Conflict of interest

The authors declare that the research was conducted in the absence of any commercial or financial relationships that could be construed as a potential conflict of interest.

## Publisher’s note

All claims expressed in this article are solely those of the authors and do not necessarily represent those of their affiliated organizations, or those of the publisher, the editors and the reviewers. Any product that may be evaluated in this article, or claim that may be made by its manufacturer, is not guaranteed or endorsed by the publisher.

## Supplementary material

Supplementary Figure Expression of MR (CD206) on MDSC and BMC. **(A, C, E, G, I, K)** One representative result from each experiment is shown. M-MDSC were gated by CD11b^+^Ly6C^+/high^Ly6G^-^ cells and PMN-MDSC were gated by CD11b^+^Ly6C^low^Ly6G^+^ cells. The percentages of MR^+^ (CD206^+^) cells among the M-MDSC **(B)** and PMN-MDSC **(D)** isolated from normal mouse. The percentages of MR^+^ (CD206^+^) cells among the M-MDSC **(F)** and PMN-MDSC **(H)** induced *in vitro*. The percentages of MR^+^ (CD206^+^) cells among the BMC which were isolated from normal mouse (in day 0) **(J)**, The percentages of MR^+^ (CD206^+^) cells among the BMC which were cultured with recombinant mouse GM-CSF in day 4 **(L)**. n=10, Data are expressed by mean ± SD.
