# Supplementary figures and images for: Effect of Angelica polysaccharide on mouse myeloid-derived suppressor cells
Source: Front Immunol. 2022 Sep 9;13:989230. doi: 10.3389/fimmu.2022.989230 (PMC9500156; doi:10.3389/fimmu.2022.989230)

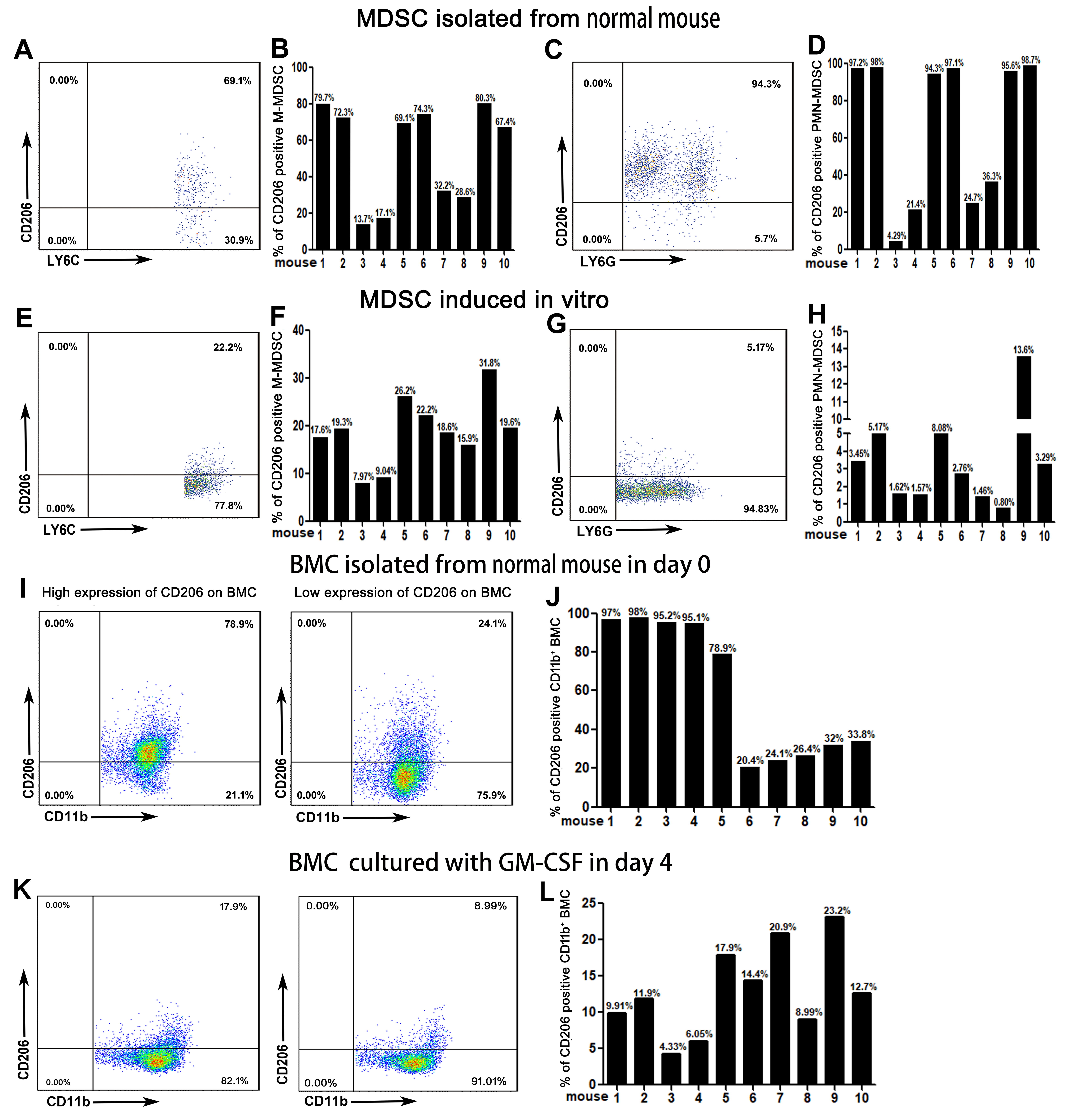

Supplement: Supplementary Figure — Expression of MR (CD206) on MDSC and BMC. (A, C, E, G, I, K) One representative result from each experiment is shown. M-MDSC were gated by CD11b+Ly6C+/highLy6G- cells and PMN-MDSC were gated by CD11b+Ly6ClowLy6G+ cells. The percentages of MR+ (CD206+) cells among the M-MDSC (B) and PMN-MDSC (D) isolated from normal mouse. The percentages of MR+ (CD206+) cells among the M-MDSC (F) and PMN-MDSC (H) induced in vitro. The percentages of MR+ (CD206+) cells among the BMC which were isolated from normal mouse (in day 0) (J), The percentages of MR+ (CD206+) cells among the BMC which were cultured with recombinant mouse GM-CSF in day 4 (L). n=10, Data are expressed by mean ± SD. [file Image_1.tif]
